# INTERSECTIONAL ENTANGLEMENT IN LIVING WITH DEMENTIA

**DOI:** 10.1093/geroni/igae098.0878

**Published:** 2024-12-31

**Authors:** Valerie Keller

**Affiliations:** Institute of Social Anthropology and Empirical Cultural Studies, Zurich, Zurich, Switzerland

## Abstract

Based on personal accounts of people living with dementia this study examines how people affected react on mechanism of societal exclusion such as pathologization, vulnerabilization and disempowerment. Although it has been called for citizenship of people affected in dementia activism and dementia studies for decades, public voices of people with dementia still remain unheard. The present study therefore examines intersectional entanglement of age and disability and highlights power structures which continue to silence people with dementia and exclude them from social participation. Furthermore, it will be outlined how people affected act at the margins not only to improve their own quality of life, but to achieve agency and to act as a social and political subject.

